# Dynamic Changes in the Global Transcriptome and MicroRNAome Reveal Complex miRNA-mRNA Regulation in Early Stages of the Bi-Directional Development of *Echinococcus granulosus* Protoscoleces

**DOI:** 10.3389/fmicb.2020.00654

**Published:** 2020-04-09

**Authors:** Yun Bai, Zhuangzhi Zhang, Lei Jin, Yongqiang Zhu, Li Zhao, Baoxin Shi, Jun Li, Gang Guo, Baoping Guo, Donald P. McManus, Shengyue Wang, Wenbao Zhang

**Affiliations:** ^1^Central Laboratory, Shanghai Skin Disease Hospital, Tongji University School of Medicine, Shanghai, China; ^2^Shanghai-MOST Key Laboratory of Health and Disease Genomics, Chinese National Human Genome Center at Shanghai, Shanghai, China; ^3^Veterinary Research Institute, Xinjiang Academy of Animal Sciences, Urumqi, China; ^4^State Key Laboratory of Pathogenesis, Prevention and Treatment of High Incidence Diseases in Central Asia, Clinical Medical Research Institute, The First Affiliated Hospital of Xinjiang Medical University, Urumqi, China; ^5^Molecular Parasitology Laboratory, QIMR Berghofer Medical Research Institute, Brisbane, QLD, Australia; ^6^National Research Center for Translational Medicine, Ruijin Hospital, Shanghai Jiao Tong University School of Medicine, Shanghai, China

**Keywords:** *Echinococcus granulosus*, protoscoleces, microRNA, transcriptome, differential expression, bi-directional development

## Abstract

**Background:**

Cystic echinococcosis is a life-threatening disease caused by the larval stages of the dog tapeworm *Echinococcus granulosus*. Protoscoleces (PSCs) of this worm have the ability of bi-directional development to either larval cysts or strobilar adult worms. However, the molecular mechanisms underlying this development process are unknown.

**Results:**

RNA and small RNAs sequencing was employed to characterize the gene and miRNA expression at 0–24 h and 7–14 days in the bi-directional development of PSCs. A total of 963 genes and 31 miRNAs were differentially expressed in the early development of PSCs to adult worms whereas 972 genes and 27 miRNAs were differentially expressed in the early development of PSCs to cysts. Pairwise comparison between the two developmental patterns showed that 172 genes and 15 miRNAs were differentially expressed at three time-points. Most of these genes were temporally changed at 24 h or 7 days. GO enrichment analysis revealed that the differentially expressed genes in early adult worm development are associated with nervous system development and carbohydrate metabolic process; whereas, the differentially expressed genes in early cystic development are associated with transmembrane transporter activity and nucleoside triphosphatase activity. In addition, miR-71 and miR-219 regulated genes are likely involved in oxidation reduction in adult worm development.

**Conclusion:**

The early stages of bi-directional development in *E. granulosus* PSCs are controlled by miRNAs and genes likely associated with nervous system development and carbohydrate metabolic process. ATP-dependent transporter genes are associated with cystic development. These results may be important for exploring the mechanisms underlying early development in *E. granulosus* providing novel information that can be used to discover new therapeutics for controlling cystic echinococcosis.

## Introduction

*Echinococcus granulosus* sensus tricto is the causative agent of CE, which is an important zoonosis with an almost cosmopolitan global distribution (McManus et al., 2003; Moro and Schantz, 2009; Wen et al., 2019). The life cycle of *E. granulosus* is complex and involves two mammalian hosts: a definitive host in which mature adult worms are produced and an intermediate host where larval cysts occur. The mature adult worm resides in the small intestine of the definitive carnivore host (dog, wolf, fox) and releases eggs which contaminate vegetation and water sources. Following ingestion by an intermediate host such as a human or sheep, the eggs hatch to release larval oncospheres which penetrate through the intestinal wall and migrate via the circulatory system to various organs (mainly the liver and lungs). In these organs, the oncospheres develop into hydatid cysts over many months and then produce PSCs which may remain in an inactive state for years. However, when a definitive host swallows the infected organs, the PSCs may be released from the cysts and develop into adult worms in the dog intestine. In contrast, if a hydatid cyst ruptures within an intermediate or human host, each released PSC is capable of developing into a secondary hydatid cyst (Zhang et al., 2005).

The bi-directional development of PSCs is a remarkable feature of *E. granulosus*. However, the mechanisms underlying this developmental process remain largely undefined. Several transcriptional and proteomic studies have identified some DEGs in the different life cycle stages of *E. granulosus* (Monteiro et al., 2010; Parkinson et al., 2012; Cui et al., 2013; Zheng H. et al., 2013). However, these genes may only be responsible for stage-specific biological processes or structural changes and may not include the genes involved in early differentiation of the parasite into either a strobilated adult tapeworm or hydatid cyst. The successful *in vitro* culture of PSCs provides an important model for studying the early development and differentiation of *E. granulosus* (Smyth et al., 1966; Smyth, 1990b; Zhang et al., 2005) to either the cystic form or adult worms triggered by bile acids. A recent study of cultured parasites identified 75 differentially expressed proteins at 24 h by the azidohomoalanine (AHA)-specific labeling method (Debarba et al., 2015), but the study provided very limited data of the associated transcriptional events occurring.

MicroRNAs are usually highly conserved throughout the animal kingdom; they are involved in embryonic development and cell differentiation of specific developmental stages. For example, lin-4 is highly expressed in the first larval stage of *Caenorhabditis elegans* and controls diverse postembryonic developmental events (Lee et al., 1993). In addition, some miRNAs are highly tissue-specific expressed and exhibit distinct temporal expression patterns in *Schmidtea mediterranea* (Palakodeti et al., 2006; Friedlander et al., 2009), *Schistosoma japonicum* (Huang et al., 2009; Hao et al., 2010), *Schistosoma mansoni* (de Souza Gomes et al., 2011), *Echinococcus multilocularis* (Cucher et al., 2015), and *Echinococcus canadensis* (Macchiaroli et al., 2015), suggesting that miRNAs play a key role in the differentiation and development of helminth worms. Although our previous study identified 42 mature miRNAs and 23 miRNAs^∗^ differently expressed in three life-stages of *E. granulosus* (Bai et al., 2015), the roles of these miRNAs in the early stages of the bi-directional development process have not been defined.

In the present study, we used NGS to profile global miRNAs and mRNAs expression during the *in vitro* culture of PSCs at multiple time points (0–24 h, 7–14 days). Bioinformatics analysis showed that nervous system development, carbohydrate metabolism, and ion transmembrane transporter may be critical in the early differentiation of PSCs into either adult worms or larval cysts. The results increase knowledge on the process of *E. granulosus* differentiation at the molecular level.

## Materials and Methods

### Collection of PSCs

Sheep livers containing hydatid cysts of *E. granulosus* (G1 genotype) were collected from a slaughterhousein Urumqi, Xinjiang, China. PSCs were collected from the cysts by aspiration, and then centrifuged at 1000 × *g* for 10 min. After washing 5 times with PBS, pH 7.4 (Zheng H. et al., 2013), the viability of the PSCs was determined by trypan blue staining (Zhang et al., 2013). Only samples of PSCs with a viability greater than 95% were utilized for further study.

### Culture of PSCs and Sample Preparation

Protoscoleces were cultured *in vitro* as described (Smyth, 1990a), with minor modifications (Zhang et al., 2005). Briefly, PSCs were digested at 37°C for 30 min with 1% (w/v) pepsin (Sigma, St. Louis, MO, United States) prepared in 0.85% (w/v) sodium chloride, pH 2.0. After the digestion procedure, the PSCs were washed three times with PBS containing antibiotics (100 IU/mL penicillin and 100 mg/mL streptomycin, Sigma, St. Louis, MO, United States) and incubated in 37°C RPMI 1640 medium (Invitrogen, San Diego, CA, United States) containing 20% (v/v) fetal calf serum (Gibco, Auckland, NZ, United States), 0.45% (w/v) yeast extract, 0.4% (w/v) glucose, 100 IU/ml of penicillin, and 100 ug/ml of streptomycin at 37°C for 30 min. A solid nutritive base was made by heating newborn calf serum at 76°C for 45 min. To obtain adult worms (strobilar development, SSD), 2000 PSCs/mL were cultured in the above medium containing 0.02% (w/v) sodium taurocholate at 37°C. To obtain cysts, PCS were cultured in the same system as SSD but without the addition of sodium taurocholate (NSD). In both cases, medium was changed every 3 days. Cultured PSCs were collected at day 1, 7, and 14 days after *on vitro* cultivation for transcriptome and microRNAome analysis. We also collected cultured PSC treated only with pepsinand collected immediately, representing the baseline of gene expression. All parasite materials were subjected to a final wash in PBS and then they were suspended in 10 volumes of RNAlater (Ambion, Austin, TX, United States), and stored at −80°C.

### RNA Isolation

Total RNA was extracted from echinococcal samples collected at the different time points using mirVana^TM^ miRNA isolation kits (Ambion, Austin, TX, United States), according to the manufacturer’s instructions. RNA concentration and purity were measured using a NanoDrop ND-1000 UV spectrophotometer (Nanodrop Technologies, Wilmington, DE, United States). RNA integrity was evaluated by an Agilent 2100 Bioanalyzer (Agilent Technologies, Palo Alto, CA, United States).

### Library Preparation and Sequencing of mRNA

About 10 μg total RNA was used for the purification of RNAs containing poly(A) using the MicroPoly(A) Purist Kit (Ambion/Applied Biosystems) according to the manufacturer’s protocols. cDNA was synthesized using the SuperScript Reverse Transcriptase II (Life Technologies, Gaithersburg, MD, United States) kit with random primers. Paired-end cDNA libraries were generated by TruSeq RNA kit (RS-122-2001, Illumina) and sequenced on the Illumina Hiseq 2500 next generation sequencing platform.

### Small RNA Sequencing

About 5 μg total RNA was used to isolate small RNAs (size 18 to 30 nt) on denaturing PAGE gels. Then, the isolated small RNAs were prepared according to Illumina’s Small RNA v1.5 Sample Preparation guide (Illumina, San Diego, CA, United States). Briefly, the small RNAs were ligated to Illumina’s small RNA 3′ and 5′ adaptors. Subsequently cDNA was synthesized by reverse transcription and amplified by PCR (12 cycles). The amplified fragments were purified by 6% (w/v) PAGE. Sequencing of the small RNA library was undertaken using the Illumina Genome Analyzer II (Illumina, San Diego, CA, United States).

### RNA-SEQ Analysis

Adaptors were trimmed from raw reads using Trimmomatic v0.35 (Bolger et al., 2014) (with the setting conditions: sliding window: 4 bp, minimum average PHRED quality: 10; minimum read length: 40bp). To obtain an accurate assembly of transcripts, we applied Trinity v20140413p1 (Grabherr et al., 2011) and PASA v2.0.2 (Haas et al., 2003) to reconstruct transcripts for every gene. For the Trinity *de novo* and genome-guided assembly (assembly number: ASM52419v1), we used the default parameters. Tophat 2.0.8 (Kim et al., 2013) was implemented to map the trimmed reads to the *E. granulosus* genome with a maximum of two mismatches. Minimum and maximum fragment sizes were set at 300 and 500 bp, respectively. The remainder of the parameters were left as default. Differential expression analysis between time points was performed using DESeq (version 1.24.0) after counting the number of reads per transcript using featureCount v1.4.6 (Liao et al., 2014). Significance was assessed as having an experiment-wide FDR < 0.05 (calculated using the Benjamini–Hochberg method) and a fold-change value >2 or <0.5.

### miRNA Analysis

Small RNA reads were first trimmed from the 5′ adapter sequence through FASTX toolkits^1^. Then, we removed low quality sequence reads and collected small RNAs ranging from 18–30 nucleotides. After removing duplicated sequences from the initial dataset, we compared all sequence tags against a database of known miRNA^2^, and profiled every annotated miRNA in each library. We discarded all sequences matching with known *E. granulosus* rRNA, tRNA, snRNAs, mRNAs, and repeat sequences in the *E. granulosus* genome. The remaining unique sequences were predicted by miRDeep 2.0.7 (Friedlander et al., 2012) and miRNA candidates were selected based on the criteria described previously (Bai et al., 2015). All known or novel mature miRNAs were counted by the quantifier module of miRDeep2 and relative expression levels were calculated by counting the numbers of respective miRNA reads normalized to the total number of annotated miRNAs (TPM) in each library. After log2 transformation of each expression value (TPM + 1), differential expression between the two developing directions was evaluated using DEGSeq. The significance value was adjusted using FDR (Benjamini–Hochberg) to correct for multiple testing.

### miRNA Target Prediction

3′-untranslational regions were determined using the pipeline described in a previous study (Macchiaroli et al., 2017). We used complete gene structures reconstructed by PASA analysis. Only sequences located at the 3′ untranslated region of predicted genes were regarded as potential 3′-UTR sequences. The 3′-UTR sequences were extracted from the *E. granulosus* genome and length distribution analysis was performed using R custom scripts. Then, two algorithms, miRanda (Enright et al., 2003) and TargetScan, were used to predict the target genes of all mature miRNAs. The miRanda Energy thresholds were set at ≤-20 kcal/mol and other thresholds used a default value (score threshold, 140; gap-opening penalty, -9; gap-extend penalty, -4).

### Functional Annotation

Novel transcripts, including alter splicing transcripts of known genes and new identified unigenes, were determined by PASA. Each alter splicing transcript was supported by at least two uniquely mapped reads. Unigene sequences were aligned to the non-redundant protein database (nr) using BLASTX (*E*-value < 10^–5^) for obtaining both protein and functional annotation information. Based on the annotations in the protein database, Blast2GO program^3^ was used to obtain GO annotations for the DEGs. Then, the hypergeometric test was used to classify the GO category, and the FDR was calculated to correct the *P*-value. KEGG pathway analysis was carried out using the KEGG Automatic Annotation Server for ortholog assignment and pathway mapping^4^. The hypergeometric test was used to assess significant pathways of enrichment.

### Validation of Differentially Expressed Genes and miRNAs by Quantitative RT-PCR

To validate the Illumina sequencing results, we randomly selected 10 DEGs and 10 miRNAs and used quantitative RT-PCR (qPCR) to determine their expression levels at the different time points. Total RNA was extracted using mirVana^TM^ miRNA isolation kits (Ambion, Austin, TX, United States) according to the manufacturer’s instructions. The RNA was reverse transcribed by PrimeScript RT Enzyme Mix I (Takara Bio, Otsu, Japan). The primers used in the qPCR analysis are listed in Supplementary Table S16. We respectively evaluated 5.8S rRNA and U6 as house-keeping small RNAs and *GAPDH*, *EIF3* and ß-actin as reference genes. Normfinder was used to find the most stable expressed house-keeping gene under the test conditions. As a result, 5.8S rRNA (*M* = 0.08) and GAPDH (*M* = 0.23) were selected as reference genes for quantitative (q)-PCR analysis. The RT reaction mixture was incubated at 37°C for 30 minutes, then at 85°C for 5 s. A control was set up at the same time with no RNA input. With the cDNA products as a template, qPCR was carried out using SYBR^®^ Premix Ex Taq^TM^ (Takara Bio, Otsu, Japan) in the StepOne Plus real-time system (Applied Biosystems, Carlsbad, CA, United States). For each qPCR, dissociation curve analysis was carried out to discriminate the specific products from the primer dimers. The CT-values were the average of three technical and three biological replicates and fold changes of miRNAs in different samples were calculated by the 2^–ΔΔCt^ method.

## Results

**FIGURE 1:**
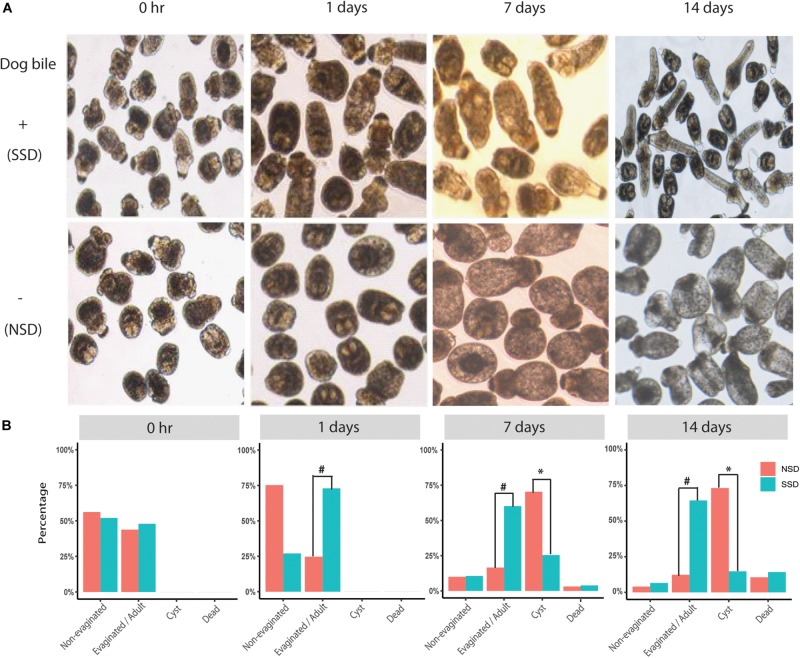
Bi-directional development of *E. granulosus* protoscoleces *in vitro*. **(A)** The structural changes in *in vitro* cultured PSC in the presence or absence of bile salt as a strobilization stimulus. **(B)** A scale bar to summarize the percentages of the different development directions, including adult worms (Evagination/Adult), hydatid cyst (Cyst), dead (Dead), and no changes (Non-Evagination). ^#^A significant increase in scolex evagination occurred at 1–7 days after stimulation with the dog bile salt (*P* < 0.001). When the PSC were cultured for 14 days, the calcareous corpuscles were much reduced and suckers and the first proglottid had formed in adult worms. ^∗^A significant increase of PSC developing to cysts at 7–14 days compared with SSD (*P* < 0.001).

We successfully used an *in vitro* culture method to promote the early development of PSCs into either adult worms of 3−5 proglottids or cystic metacestode cysts (up to 2 cm in size) (data not shown). The bi-directional development of PSCs in the culture system (with or without dog bile) is shown in Figure 1. Increased scolex evagination and the production of numerous calcareous corpuscles was observed within the first 3−7 days of cultivation in culture medium containing bile salt (sodium taurocholate). However, after 14 days culture, the calcareous corpuscles had disappeared; lateral excretory canals were conspicuous and a genital rudiment was present denoting the formation of the first proglottid. In culture medium without the bile salt, the PSCs developed into cysts; these developing PSCs contained calcareous corpuscles in the first 3−7 days as was evident during adult worm development. Cysts were then subsequently formed (Figure 1).

### Summary of RNA and Small RNA Sequencing and the Alignment of Reads

We compared the transcriptomes and microRNAomes of the cultured *E. granulosus* PSCs harvested at 1, 7, and 14 days after culture in medium with (early adult worm development) or without (early cystic development) dog bile salt. A total of 394.92 million and 210.72 million Illumina raw reads were respectively generated from the transcriptomic and microRNAomic libraries. The raw reads were deposited in the National Center for Biotechnology Information Sequence Read Archive under the accession number PRJNA610943. After removing low-quality sequences, adaptor contaminants and small reads (less than 40 bp in RNA-seq and 18 nts in small RNA-seq), we obtained 352.59 million and 163.95 million clean reads, respectively. Of these reads, an average of 65.63% and 51.12% could be mapped to the *E. granulosus* genome (Table 1).

**TABLE 1:** Data summary of the *E. granulosus* transcriptome and microRNAome.

**Samples ID**	**RNA Sequencing**			**Small RNA Sequencing**		
	
	**Total Clean Reads**	High Quality Reads Genome Mapped (%)*	**High Quality reads Transcripts mapped (%)^#^**	**Total Clean Reads (18−30 nt)**	High Quality Reads Genome Mapped (%)*	**High Quality Reads miRNAs Mapped (%)^#^**
0 h	56,805,518	41,477,149 (73.02%)	27,483,646 (66.26%)	24,185,579	13,168,238 (54.45%)	7,249,946 (55.05%)
**NSD**						
24 h	45,865,714	27,534,887 (60.03%)	20,807,190 (75.57%)	22,664,542	13,374,352 (59.01%)	8,162,254 (61.03%)
7 days	47,387,288	30,641,662 (64.66%)	21,343,889 (69.66%)	22,053,979	12,312,969 (55.83%)	6,996,764 (56.82%)
14 days	55,597,998	38,266,289 (68.83%)	28,976,806 (75.72%)	22,113,695	11,027,824 (49.87%)	5,184,590 (47.01%)
**SSD**						
24 h	55,037,642	33,752,124 (61.33%)	19,137,393 (56.70%)	21,173,443	10,502,453 (49.60%)	3,546,522 (33.77%)
7 days	48,910,972	28,462,253 (58.19%)	18,073,569 (63.50%)	26,331,190	9,608,894 (36.49%)	5,773,972 (60.09%)
14 days	42,987,048	31,534,216 (73.36%)	23,182,955 (73.52%)	25,430,586	13,366,873 (52.56%)	6,859,834 (51.32%)

NSD, PSC without strobilization stimulus (no bile salt added); SSD, PSC with strobilization stimuli (bile salt added). * The percentages were calculated by dividing the counts of total clean reads. ^#^The percentages were calculated by dividing the counts of high quality reads genome mapped.

Due to the absence of sufficient information about alternative splicing isoforms and 5′ and 3′ untranslated regions (UTR) in current *E. granulosus* annotations, we performed both a *de novo* as well as a genome-guided assembly (ASM52419v1) to reconstruct the gene transcripts and then combined the outputs using the PASA pipeline. There were respectively 113,141 and 93,584 transcripts identified using these two assembly methods (Supplementary Table S1). We further combined them with known genome annotations, and identified 185 of 11,329 predicted genes that merged with neighboring genes. Furthermore, a total of 5,460 genes were re-annotated gene constructions. Among these, 3,219 genes were determined to have alternative splicing events, and 4,795 and 4,859 genes were found containing 5′-UTRs and 3′-UTRs, respectively. Supplementary Figure S1 shows the length distribution of the annotated *E. granulosus* 3′-UTR sequences. A major proportion (90.44%) of the 9,839 3′-UTRs was shorter than 4,000 nucleotides. The median length of the total set of 3′-UTRs was 1,027 nucleotides and the average GC content was 42.45%. We then mapped the high quality sequence reads to the *E. granulosus* transcriptome. The alignment summary metrics are shown in Table 1. After elimination of ambiguous sequence matches, more than 18 million reads were mapped in each library. The number of sequence reads mapped per gene varied from one to >740,000.

For miRNA sequencing, we discarded all known non-coding RNAs, such as rRNA, tRNA, snoRNA, repeat-associated RNA, and degraded fragments of mRNAs. The remaining 70.82 million high quality sequences were used to search for both known and novel miRNAs. To date, there are 111 miRNA precursors of *E. granulosus* identified in the miRBase database. They could encode 218 mature miRNAs or miRNA stars sequences. By deep sequencing, we found that more than 40% high quality sequences were matched to the known miRNA precursors in each library (Supplementary Table S2). A total of 167 known mature miRNAs or miRNA stars were identified in the present study. miR-71-5p and miR-1-3p were the most abundant in all libraries. After using miRDeep2 to predict novel miRNA precursors, we identified 7 miRNA candidates (Supplementary Table S3). Among these, the mature sequence miR-10450b sequence was highly consistent with miR-10450a in the 5′ arm, and these were classified into the same miRNA family. None of the others showed homology with any other metazoan miRNAs and were thus defined as *E. granulosus* specific miRNAs.

### Transcriptomic Changes in Early Development of PSC

To identify DEGs in PSCs during early bi-directional development, we compared the transcriptomes of PSCs treated with pepsin and cultured for 24 h and 7–14 days in the presence or absence of dog bile salt with the baseline which is gene expression in the PSCs treated only with pepsin and collected immediately. As shown in Table 2 and Supplementary Table S4, 963 DEGs were differentially expressed in the PSCs culture with bile salt strobilisation stimulus (SSD), including 579 genes that were up-regulated and that were 622 genes down-regulated. In PSCs developing cysts [i.e., without bile salt strobilisation stimulus in the culture medium (NSD)], 972 DEGs were identified, including 612 up-regulated genes and 558 down-regulated genes (Supplementary Table S5).

**TABLE 2:** Summary of the counts of differentially expressed genes over time in the two developmental (i.e., cystic or strobilar) directions.

	**24 h vs. 0 h**	**7 days vs. 0 h**	**14 days vs. 0 h**	**7 days vs. 24 h**	**14 days vs. 7 days**	**Sum**
**PSC without strobilization stimulus (NSD)**						
Total DEG	276	328	276	352	344	972
DEG (log_2_ fold-change >1.0)	110	188	161	250	151	612
DEG (log_2_ fold-change <−1.0)	166	140	115	102	193	558
**PSC with strobilization stimulus (SSD)**						
Total DEG	320	361	349	272	334	963
DEG (log_2_ fold-change >1.0)	95	246	156	193	88	579
DEG (log_2_ fold-change <−1.0)	225	115	193	79	246	622

*DEG, differentially expressed genes. The statistical comparison was determined by DESeq. Log_2_ fold-change cutoffs of +1.0 and −1.0.*

**FIGURE 2:**
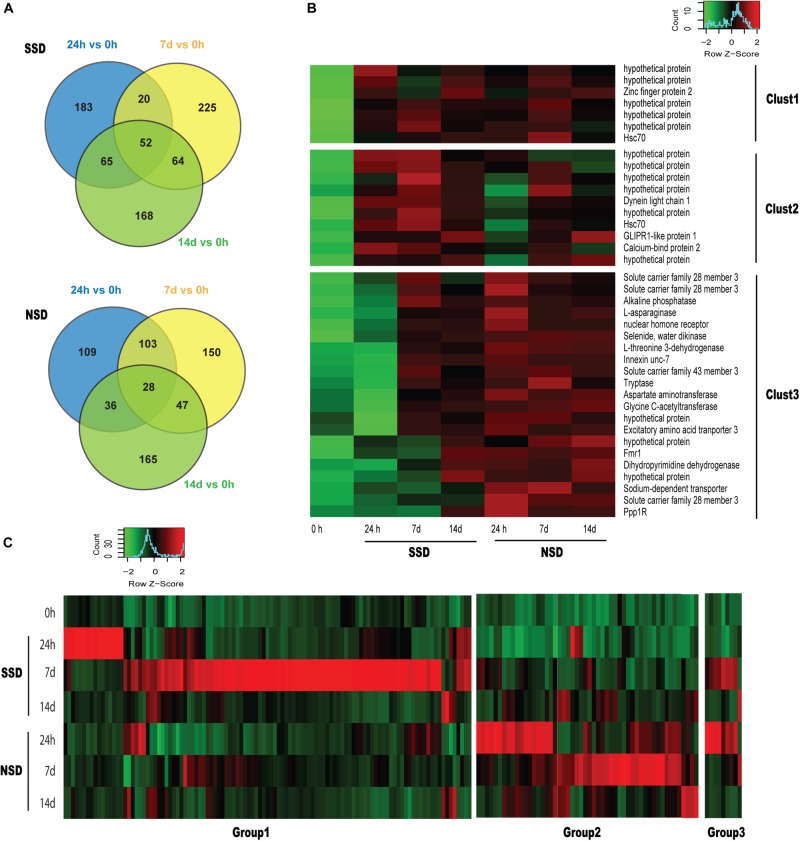
Dynamic transcriptomic changes over time. SSD: *in vitro* culture of PSC with bile salt strobilization stimulus; NSD: *in vitro* culture of PSC without bile salt strobilization stimulus. **(A)** Venn diagram showing the number of overlapping differentially expressed genes (DEGs) across pairwise comparisons at each time point compared to the baseline (0 h). **(B)** Heat maps of sequencing data from 38 overlapping DEGs identified in Figure 1A. Clust1 represents 7 DEGs which were significantly increased in the both SSD and NSD. Clust2 and Clust3 represent 10 and 21 DEGs, respectively, which were specifically and consistantly over-expressed in SSD and NSD. A *P*-value < 0.05 and a fold-change >2.0 were used as the threshold criteria to define significant differences. **(C)** Heat maps showing 172 DEGs which were specifically over-expressed at SSD or NSD (118 in the SSD and 63 in NSD). Groups1 and 2 respectively represent 109 and 54 DEGs which were only up-regulated in SSD or NSD. Group 3 includes 9 DEGs which were simultaneously over-expressed in SSD and NSD.

When comparing each time point (24 h and 7–14 days) to the baseline (0 h), we identified 52 DEGs (17 up-regulated and 35 down-regulated) in SSD and 28 DEGs (28 up-regulated and 0 down-regulated) in NSD which are differentially expressed at all three time points (Figure 2A). Based on the functional annotations of the up-regulated genes, most of the DEGs in SSD were characterized as hypothetical protein with those in NSD belonging to transporter, aminotransferase and protease (Figure 2B).

We also horizontally compared the expression profiles between the two developmental patterns at the different time-points. A total of 537 DEGs were found in SSD or NSD. After removing the non-differentially expressed genes by comparison with the baseline (0 h), we identified 172 DEGs in the two developmental patterns (118 genes were up-regulated in SSD and 63 genes were over-expressed in NSD; Figure 2C and Supplementary Table S6). Most of the DEGs were temporally changed, being mainly differentially expressed at 24 h or 7 days. There were 22 genes with increased expression at 24 h after the stimulation with bile acid and 97 genes were over-expressed at 7 days. A similar trend was also observed in NSD; there were 29 and 34 genes up-regulated at 24h and 7 days, respectively. Finally, only 6 DEGs were changed at 14d in both SSD and NSD. These results suggest that the early stage is an important time point guiding the early developmental direction of PSCs. Moreover, as shown in Figure 2C, we observed that 9 DEGs were changed in both SSD and NSD with expression changes at the different time-points, indicating that even with the same gene, its temporal and spatial pattern could determine the early developmental direction of PSCs. We used qPCR to validate the expression of 10 randomly selected DEGs of which 8 were shown to be in accordance with the expression profiles detected by the RNA sequencing (Supplementary Table S7).

### Gene Function Analysis of DEGs During the Early Development of PSCs

**FIGURE 3:**
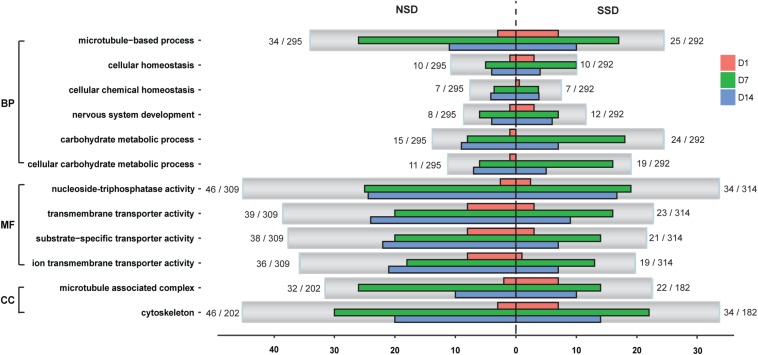
Classification of GO annotations. The *X*-axis indicates the number of genes in a sub-category and the *Y*-axis indicates the GO sub-categories. D1, *in vitro* culture for 24 h; D7, *in vitro* culture for 7 days; D14, *in vitro* culture for 14 days. SSD, *in vitro* culture of PSC with bile salt strobilization stimulus; NSD, the *in vitro* culture of PSC without bile salt strobilization stimulus; BP, biological processes; MF, molecular functions; CC, cellular components.

We conducted GO functional analysis of the up-regulated and down-regulated DEGs in SSD and NSD, with 52.0 and 64.6%, respectively, of DEGs assigned a GO term. Further enrichment analysis revealed significant over-representation of 25 terms in SSD (10 from up-regulated genes and 15 from down-regulated genes) and 25 in NSD (23 from up-regulated genes and 2 from down-regulated genes) (Supplementary Table S8). After comparing these between SSD and NSD, we identified 8 GO terms simultaneously existing in the two development directions, namely cellular homeostasis (GO: 0019725), cellular chemical homeostasis (GO: 0055082), cellular ion homeostasis (GO: 0006873), microtubule-based process (GO: 0007017), microtubule associated complex (GO: 0005875), cytoskeleton (GO: 0005856), cytoskeleton part (GO:0044430) and microtubule cytoskeleton (GO: 0015630) (Figure 3 and Supplementary Table S9); these were more prominent in NSD.

Additionally, we observed some GO terms were specifically enriched in SSD or NSD. For example, comparing the NSD, more up-regulated genes in SSD were associated with nervous system development (GO: 0007399), carbohydrate metabolic process (GO: 0005975) and cellular carbohydrate metabolic process (GO: 0044262). Meanwhile, there were 15 “Molecular Function” terms more prominent in the up-regulated genes of NSD than in SSD (Supplementary Table S9). Most of these are related to ion transmembrane transporter activity (GO: 0015075), substrate-specific transporter activity (GO: 0022857) and nucleoside-triphosphatase activity (GO: 0017111). Of note, we also observed that some biological processes had obvious stage-specificities. As shown in Figure 3, there were 18 over-expressed genes related to carbohydrate metabolic process (GO: 0005975) in SSD at 7d. This number was significantly higher than in NSD (8 genes) at the same time-point. In the GO terms of ion transmembrane transporter activity (GO: 0015075) and substrate-specific transporter activity (GO: 0022857), clear increases were evident in the amounts of up-regulated genes in NSD at 14d.

### Expression Changes in miRNAs Over Time

We compared the expression profiles of miRNAs across the different stages of early bidirectional development. A total of 31 and 27 mature miRNAs, respectively, exhibited statistically significant changes (a threshold of relative expression >50, correct *P*-value < 0.01 and fold-change >2.0) in at least one of the three time-points during SSD and NSD (Supplementary Table S10). Further clustering of these miRNAs, based on similar expression patterns, identified 5 clusters in each development direction (Supplementary Figure S2 and Supplementary Table S11). 7 days was the stage with the most significant expression change among the three time-points. There were, respectively, 13 up-regulated miRNAs (cluster 1) and 8 down-regulated miRNAs (cluster 2) identified at this time-point of SSD. A similar pattern was evident in NSD, where cluster 1 and cluster 2 respectively represented 9 and 7 specifically up- and down-regulated miRNAs. Additionally, the 24 h culture time point is also an important regulatory period in PSC development. There were respectively 5 miRNAs (cluster 3) and 3 miRNAs (cluster 4) that were specifically over expressed in SSD and NSD. In addition, we found that some miRNAs were consecutively differentially expressed at multiple stages, such as cluster 4 and cluster 5, respectively, which contained 2 and 3 miRNAs for which expression increased and decreased throughout early development in SSD. Meanwhile, cluster 5 consisted of miR-9 and miR-4989 that were strongly up-regulated in NSD at 7–14 days.

**FIGURE 4:**
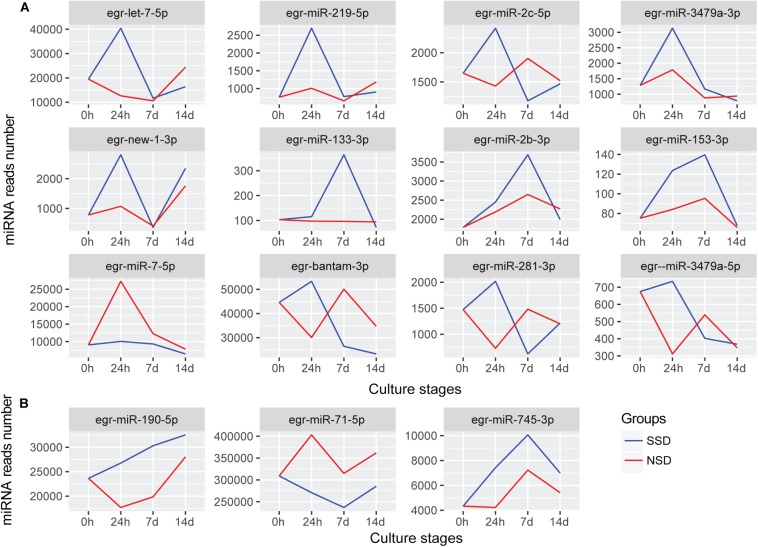
miRNAs with different expression patterns between SSD and NSD. **(A)** The differentially expressed miRNAs with different expression patterns. **(B)** The miRNAs were differential expressed throughout the three time-points. The blue line indicates the expression levels of candidate miRNAs during *in vitro* culture in the presence of the bile salt as strobilization stimulus (SSD). The red line indicates the expression levels of candidate miRNAs during the *in vitro* culture without the bile salt strobilization stimulus (NSD). The *Y*-axis indicates the relative expression levels of each miRNA.

After further comparing the expression patterns of each miRNA, we identified 12 differentially expressed miRNAs between SSD and NSD (Figure 4A). Additionally, there were 3 miRNAs (miR-190-5p, miR-745-3p, and miR-71-5p) having the same trend throughout the three time-points of development, although these differences did not reach the 2-fold threshold (Figure 4B). Among these miRNAs, 10miRNAs (let-7-5p, miR-133-3p, miR-153-3p miR-219-5p, miR-3479a-3p, miR-2b-3p, miR-2c-5p, new-1-3p, miR-190-5p, and miR-745-3p) were significantly up-regulated in SSD, and two miRNA (miR-7-5p and miR-71-5p) were specifically over-expressed in NSD. A clear characteristic of time-dependent expressions was observed in these miRNAs. For example, let-7-5p, miR-219-5p, miR-3479a-3p, miR-2c-5p, and new-1-3p were specifically over expressed at 24 h, while miR-2b-3p and miR-133-3p were only increased at 7 days. These consistent differences also imply that these miRNAs may be associated with the direction of early development of a PSC into either an adult worm or a secondary hydatid cyst. Finally, we used stem-loop real-time quantification RT-PCR to examine the expression of 10 randomly selected miRNAs of which 9 were shown to be in accordance with the expression profiles detected by the Illumina sequencing (Supplementary Table S12).

### Target Gene Prediction

To evaluate the biological functions of the 15 differentially expressed miRNAs, we predicted putative target genes using the Miranda program. Based on our 3′-UTR results in the transcriptome, we found 3,601 genes possibly targeted by 242 mature miRNAs and star sequences. Among these, a total of 971 genes may be regulated by the differentially expressed miRNAs (Supplementary Table S13). We also used the worm TargetScan to validate our predictions. As shown in Supplementary Table S13, all targets of the differentially expressed miRNAs were confirmed except for one novel miRNA which was not found in the same miRNA family in the TargetScan database. Further enrichment analyses on the predicted targets, indicated that these target genes are more abundant in the GO term of nucleotide binding (adjust *P* = 0.020, *n* = 162, Supplementary Table S14); these include cytoplasmic polyadenylation element-binding protein 1 (EG_00812), Titin (EG_02049), Casein kinase I alpha (EG_04711), Heat shock cognate 70 kDa protein (EG_08863), Tyrosine-protein kinase Srms (EG_10535), and Fyn (EG_09408), some of which have been shown to be associated with germline development (Jin et al., 2001), cell asymmetric division (Banerjee et al., 2010), and adaptation processes of parasites in the host environment (Yang et al., 2012). Moreover, we also determined that some target genes, such as let-7, bantam and miR-71, regulated by the differentially expressed miRNAs may be involved in the MAPK signaling (PATH: ko04010) and Wnt signaling pathways (PATH: ko04310) (Supplementary Figure S3). These results are consistent with a previous study (Macchiaroli et al., 2017), suggesting that these miRNAs maybe involved in *E. granulosus* development.

Finally, we observed a significant enrichment of DEGs associated with oxidation reduction process. As shown in Supplementary Table S15, there were 10 DEGs differentially expressed in this process, including Glyceraldehyde-3-phosphate dehydrogenase (EG_02307), NADH dehydrogenase 1 beta subunit 9 (EG_03558), Ferritin 1 (EG_08965), Protein yfeX (EG_07721) and Heat shock cognate 70 kDa protein (EG_09244, EG_09649). All of these genes were clearly up-regulated at 7 days in the SSD. Notably, some differentially expressed miRNAs between the SSD and the NSD may also regulate these targets. For example, a higher expression level of miR-71-5p was observed at baseline than at 7 days, yet its target gene heat shock cognate 70 kDa protein *HSC70* (EG_09649), was up-regulated at 7 days. Moreover, at 24 h and 7 days of SSD, we also observed a significant negative correlation between the expression of miR-219-5p and its putative target gene yfeX (EG_07721).

## Discussion

The bi-directional development of PSCs is a striking feature of the biology of *E. granulosus* (Zheng H. et al., 2013). In the presence of bile salt, a PSC can differentiate in a sexual direction to form a mature adult worm in the dog gastrointestinal tract. However, if a hydatid cyst ruptures within the intermediate or human host, each released PSC is capable of differentiating asexually into a new hydatid cyst. These *in vivo* developmental patterns can be replicated *in vitro* and the culture of PSCs provides a unique opportunity to determine how the gene pathways are modulated in these diverse environments. In the present study, we used NGS to study the transcriptional dynamics of PSCs cultured in the presence or absence of dog bile acid at three *in vitro* culture time points (24 h and 7–14 days). A total of 5,460 genes were re-annotated using the gene constructions based on the ESTs of genome-guided assembly. Among these, 3,219 of 10,044 detected genes underwent alternative splicing (32.0%). This ratio is very close to the proportions recorded for *Dictyocaulus viviparus* (34.6%) (Cantacessi et al., 2010) and *Cooperia oncophora* (33.1%) (Heizer et al., 2013), but is significantly higher than that reported for *C. elegans* (17%) in WormBase (Yook et al., 2012). This result is consistent with observations of parasitic nematode transcriptomes (Abubucker et al., 2014), which may be due to a reduction in the number of functional genes in parasite genomes (Zheng H. et al., 2013) or the increased genomic complexity that may be required to interact with multiple hosts/vectors (Abubucker et al., 2014).

Further expression pattern analysis of the *E. granulosus* transcriptome revealed that highly temporal gene expression was evident in both SSD and NSD. Only 52 and 28 DEGs, respectively, were found to have consistent expression changes at all three time-points. Although there are no reports of the dynamic changes in gene expression during PSC development, some stage-specific changes in morphology (Thompson, 2017) indicated that the study of PSC development needs to simultaneously consider temporal cues and environmental conditions. Through the comparison of DEGs between SSD and NSD, we found that most of the DEGs were detectable at the 24 h and 7 day culture points. This phenomenon suggests that the early developmental stage is critical for triggering the PSCs to differentiate either into an adult worm or a secondary cyst. Key regulatory genes that determine the developmental direction may be differentially expressed at this time. For example, neurogenic locus protein delta, an important ligand for the Notch receptor, is expressed at the early embryonic stage of *Drosophila* to regulate the correct separation of neural and epidermal cell lineages (Haenlin et al., 1994). In the present study, its gene homolog in *E. granulosus* was specifically over-expressed in SSD at 7d culture, suggesting that it may promote the differentiation and growth of the early nervous system in the PSC as it develops to an adult worm.

### Nutrition and Energy Metabolism in the Early Stages of the Bi-Directional Development of PSCs

Nutritional metabolism is critical for cellular and organismal development. Usually, parasites need to take up considerable amounts of nutrients from the host to maintain their own growth and reproduction. *E. granulosus*, because of multiple hosts in its life-cycle, is confronted by a range of complex host environments, such as the liver or lungs in intermediate hosts or the small intestine in definitive hosts. This diversity has led these worms to modify their energy metabolism to adapt to the different hosts and environments. We showed previously that *E. granulosus* has complete pathways for glycolysis, the tricarboxylic acid cycle and the pentose phosphate pathway, but lacks the capability for *de novo* synthesis of pyrimidines, purines and most amino acids (except for alanine, aspartic acid and glutamic acid) (Zheng H. et al., 2013); indicating that *E. granulosus* has to rely on obtaining some essential nutrients directly from its mammalian hosts. In addition, more genes associated with aerobic metabolism were observed to be up-regulated in SSD, and these included phosphoglucomutase, glucokinase, fructose-bisphosphate aldolase, pyruvate dehydrogenase and citrate synthase. This increased level of expression can be interpreted in two ways. Firstly, all nutrients taken in by the adults of *E. granulosus* are absorbed through the tegument. However, due to the limitations of its small volume and surface area, the adult worms have to use aerobic glucose metabolism to yield a higher level of energy than anaerobic glycolysis. It has been reported that adults of *E. granulosus* worms tend to produce more acetate and succinate and less ethanol in the small intestine of the definitive host (Constantine et al., 1998). Secondly, compared with the metacestode cyst, the adult worm of *E. granulosus* has a remarkably complex nervous system (Koziol et al., 2013). This current study has revealed that many neuro-developmental related genes were over-expressed in SSD. Although there are no reports that have considered the relationship between nervous development and carbohydrate metabolism in *E. granulosus*, studies on another cyclophyllidean cestode, *Mesocestoidescorti*, showed that the distribution of glycogen changed according to the distribution of nerve codes during its development from a larva to an adult worm (Cabrera et al., 2010); this implies that nervous system development may utilize carbohydrate metabolism to provide the necessary energy requirements.

Glycans are important components of a wide variety of microbial pathogens, including viruses, bacteria, and parasites, including *E. granulosus*. They are widely distributed at hydatid cyst membranes and in PSCs (Khoo et al., 1997), and have been shown to control host complement activation (Diaz et al., 1999). In the present study, we found that the levels of some enzymes involved in the synthesis of polysaccharides, including beta-1,3-N-acetyl-glucosaminyltransferase, UDP-glucose and 6-dehydrogenase, were increased in SSD, suggesting that glycans may not only protect the cystic stage from immune attack, but may also assist the adult worms in attaching to its definitive host. Furthermore, lectin fluorescence assays have shown that the glycans are widespread in the tegument and parenchyma components, including the reproductive system in adult *E. granulosus* (Casaravilla et al., 2003).

### Ion Channel and Nutrient Transporters in the Cyst

Host-parasite crosstalk at the cellular and molecular levels is essential for *E. granulosus* survival and development. Our previous genomic study identified two different mechanisms of material exchange occur simultaneously in this tapeworm, including microtubule-dependent passive diffusion and ATP-dependent active transport (Zheng H. et al., 2013). In the present study, we observed that the up-regulated genes in the microtubule-based process were significantly enriched in the both SSD and NSD, a result suggesting that passive diffusion may be involved mainly in the provision of some critical metabolites for worm survival. Furthermore, we also noted a significant increase in the expression levels of ATP-dependent transporter genes in NSD, suggesting the more actively growing cyst requires more essential nutrients from its host.

Additionally, we also found that many calcium (Ca^2+^) transporters were over-expressed in NSD. In general, Ca^2+^ gains access into cells across the plasma membrane through voltage-gated Ca^2+^ channels. In order to maintain a very large transmembrane electrochemical gradient, the cell has to remove Ca^2+^ through ATP-dependent Ca^2+^ transporters to maintain low concentrations of Ca^2+^ intracellularly (Carafoli, 1991). A number of studies have shown that praziquantel can act directly or indirectly on Ca^2+^ channels and effectively kill adult *Schistosoma* and *Echinococcus* worms (Andersen et al., 1981; Richards et al., 1988; Xiao et al., 1994; Greenberg, 2005; Wei et al., 2005). These findings not only suggest that Ca^2+^ homeostasis is critical to the survival and development of *E. granulosus*, but also imply that interfering with Ca^2+^ transport may be a novel alternative approach for humancystic echinococcosis treatment. A recent study has reported that low doses of tamoxifen reduced the survival of *E. granulosus* both *in vitro* and *in vivo* (Nicolao et al., 2014). The drug may induce increased release of ATP-dependent Ca^2+^ from the endoplasmic reticulum and disrupt Ca^2+^ homeostasis (Bollig et al., 2007).

Albendazole and mebendazole are proven effective drugs for the treatment of CE. These compounds not only inhibit the development of PSCs and germinal cells in a dose-dependent manner *in vitro*, but they also reduce cyst weight after oral application in infected mice (Liu et al., 2015). In the past, it was believed that benzimidazole treatment efficacy resulted mainly from selectively inhibiting the synthesis of microtubules in parasitic worms, and destroying extant cytoplasmic microtubes in their intestinal cells, thereby blocking the uptake of glucose and other nutrients (Schmidt, 1998). However, a recent study found that mebendazole can significantly inhibit the AST enzyme activity of hydatid cysts (Naderloo et al., 2016). Our present study also showed that the expression levels of AST and asparaginase were significantly increased during the process of NSD, suggesting that albendazole or mebendazole not only reduce the uptake of glucose and other nutrients, but also interfere with the synthesis of some essential amino acids.

### miRNAs of *E. granulosus* as Novel Therapeutic Targets for Parasite Control

In the present study, we compared the expression profiles of miRNAs in the early stages of the bi-directional development of *E. granulosus*. We identified 15 miRNAs with differentially expressed patterns between SSD and NSD. These miRNAs were differentially expressed at either some specific time points or during the whole development process and may directly determine the developmental direction of PSC. Accordingly, effective inhibition of these miRNAs as novel targets may represent an effective new avenue targets for the treatment of echinococcosis.

One of the most abundant miRNAs in all the early PSC development libraries was miR-71-5p. In *C. elegans*, miR-71may enhance longevity through the negative regulation of PHA-4/FOXA and DAF-16/FOXO (Boulias and Horvitz, 2012; Smith-Vikos et al., 2014). Furthermore, miR-71 is involved in neuronal development in nematodes (Hsieh et al., 2012) and in the biological function of neoblasts, which are the only known cell type in the Platyhelminthes with the ability to undergo mitosis (Zheng Y. et al., 2013). We showed that miR-71 was significantly highly expressed in NSD and down-regulated in SSD, and suggest that its targets may be involved in the regulation of the MAPK and Wnt signaling pathways in SSD. A recent study proposed that MAPK may be a therapeutic target in *E. granulosus* through the suppression of MKK3/6 and MEK1/2 (Zhang et al., 2019). Furthermore, some Wnt-pathway related proteins, such as the frizzled receptor eg-fz4, have been shown to be highly expressed in PSC cultured *in vitro* toward adult maturation, and that they may be involved in early development of the nervous system and gut-derived organs (Pan et al., 2006; Dezaki et al., 2016; Zidek et al., 2018). These results are consistent with a previous study (Macchiaroli et al., 2017), and suggest that the down-regulation of miR-71 in SSD might trigger the MAPK and Wnt pathways in growth and development.

In addition, many components, including proteins, small molecular compounds and miRNAs, have been shown to be involved in the interactions between the mammalian host and *E. granulosus*; they can not only promote the growth of parasites, but also affect the immune response of the host (Zheng Y. et al., 2013). A study of comparative proteome profiling in *E. granulosus* identified many host proteins in human hydatid cyst fluid, indicating that the hydatid cyst is permeable allowing constant protein exchange between the metacestode and affected host tissue (Zeghir-Bouteldja et al., 2017). It has been shown that nematode miR-71 can be released into the host via exosomes (Buck et al., 2014). The transfection of *E. multilocularis* miR-71 mimics on murine macrophage cells significantly repressed the production of NO after treatment with lipopolysaccharide (LPS) (Zheng et al., 2016). The production has been shown to play an essential role in limiting the extent of infection and effecting immunosuppressive anti-Echinococcus immune responses (Zheng, 2013). These results suggest that the up-regulation of miR-71 in NSD may result in the interference of host innate immunity following its the secretion from the parasite to the host.

let-7 is an the important member of the miRNA family first identified in *C. elegan* (Sokol, 2012). Some studies have suggested that it can act as a developmental switch to control the transition from larva to adult (Reinhart et al., 2000). We previously showed that *E. granulosus* let-7 could complementary bind to the VDR 3′-UTR sequences, which may mediate a negative feedback loop controlling the bile acid signaling pathway in the early development of PSCs (Bai et al., 2015). In the present study, we observed a significant up-regulation at 1 days of SSD, further emphasizing that let-7 may regulate the expression of VDR and promote adult development in *E. granulosus*. Recently, an *in vitro* study showed that the exposure of albendazole to cultured PSCs significantly reduced let-7 expression and inhibited the differentiation of microcyst formation (Mortezaei et al., 2019); in contrast, higher let-7 expression and a lower growth inhibitory rate were observed in segmented worms, suggesting that let-7 may be an essential developmental regulator in the bi-directional development of *E. granulosus*.

Finally, although the synthesis of many *E. granulosus* proteins followed the expression of their corresponding transcripts, we observed a weak correlation between the transcriptome and newly synthesized proteins. This inconsistence may be ascribed to the following reasons: (1) the half-lives of the various proteins are very different. The level of a given protein is not only governed by protein synthesis, but is also dependent on the rates of degradation in different conditions; (2) some minor differences in RNA abundance may result in significant changes in the level of protein synthesis for a number of proteins. (3) post-transcriptional editing of miRNAs may also contribute to the differences evident between the transcriptome and the proteome.

In summary, the successful *in vitro* culture of PSCs represents an important model for studying the early development of *E. granulosus*. Using this model, we first characterized the dynamics of the mRNA transcriptome of cultured PSCs in the presence or absence of bile salt. We observed the bile salt stimulated significant effects on gene expression. The major gene expression changes occurring during strobilar development were related to carbohydrate metabolism and nervous system development. Additionally, we showed that the transcription of ion channel and ion transmembrane transporters was significantly increased during early cystic development. This feature supports the current view that most of the drugs for the effective treatment of CE target ion transmembrane transport in *E. granulosus*.

Furthermore, miRNA analysis revealed that 15 miRNAs had differentially expressed patterns between the two developmental (strobilar or cystic) directions. Among of these, miR-71 was highly expressed in *E. granulosus*, but not expressed in the mammalian host, and may thus be assessed in future as a candidate target for diagnositic marker for CE. Recently, a primary cell cultures of *E. multilocularis* found that 2’-O-methyl modified anti-miR-71 led to a significantly reduced level of miR-71 and abnormal development of PSCs (Perez et al., 2019). This outcome suggests that antisense oligonucleotides of miRNAs may be considered as a future clinical intervention strategy against echinococcosis. Therefore, understanding the transcriptional and regulatory characteristic of PSCs under the different *in vitro* culture conditions may be helpful in exploring the mechanisms governing cystic or adult worm development in *E. granulosus*. This information provides a new avenue to develop new interventions and therapeutics for the control of CE.

## Data Availability Statement

High throughput sequencing data have been submitted to the NCBI Sequence Read Archive (SRA) under accession number PRJNA610943.

## Author Contributions

WZ and SW coordinated the project. ZZ, LZ, BS, JL, and WZ participated in the *E. granulosus* sample preparation and RNA extraction. SW, YB, LJ, and YZ directed and performed the mRNA and small RNA sequencing. YB performed the sequence alignment and the novel miRNAs prediction, annotation, and expression analysis. YB, GG, BG, and LJ carried out the quantitative RT-PCR. SW, WZ, and DM commented on the mRNA and small RNA sequencing and analysis. YB, DM, SW, and WZ wrote the manuscript.

## Conflict of Interest

The authors declare that the research was conducted in the absence of any commercial or financial relationships that could be construed as a potential conflict of interest.

## Footnotes

1 http://hannonlab.cshl.edu/fastx_toolkit/index.html

2 http://www.mirbase.org/, release 22.0

3 http://www.blast2go.com/

4 http://www.genome.jp/tools/kaas

## Supplementary Material

FIGURE S1 Length distribution analysis of *E. granulosus* 3′-UTRs. The figure summarizes the length distribution of *E. granulosus* 3′-UTR. A major proportion of the 9,839 3′-UTRs (90.44%) were shorter than 4,000 nucleotides. The median length of the total set of 3′-UTRs was 1,027 nucleotides and the average GC content was 42.45%.

FIGURE S2 Clustering of differentially expressed miRNAs during the *in vitro* development of PSCs. The differentially expressed miRNAs were clustered using the K-means method. Expression values were normalized and scaled between −1.0 and 1.0 (*Y*-axis). The *X*-axis indicates the time-points when RNA was subjected to transcriptome sequencing. A description of the pattern of expression belonging to each cluster is shown for each panel.

FIGURE S3 Predicted targets of the differentially expressed miRNAs within the context of selected signaling pathways. **(A)** Wnt signaling pathway; **(B)** MAPK signaling pathway. Pink boxes represent gene orthologs present in *E. granulosus*. miRNAs that target genes in these pathways are shown in red.

TABLE S1 Assembly of RNA-seq and alternatively spliced genes estimates. The table summarizes the counts of genes detected in RNA-seq. The percentages were calculated by dividing the counts of detected genes in all samples; AS, Alternatively splicing.

TABLE S2 Raw data filtration and distribution of sequenced small RNAs across different categories. All small RNA reads were aligned to the reference *E. granulosus* genome and related RNA sequences using bowtie. One mismatch was permitted. The percentages were calculated by dividing the counts of reads matched to the genome. SSD: PSC with bile salt strobilization stimulus; NSD: PSC without bile salt strobilization stimulus.

TABLE S3 All predicted novel miRNA precursors in *E. granulosus*. The table lists the sequences and MFE of candidate novel miRNAs in *E. granulosus*. If a miRNA candidate has multiple loci in the *E. granulosus* genome, we distinguish this candidate using a different number at the end of the miRNA names. Score, score of mirDeep; MFE, minimum free energy; Pre, miRNA precursors.

TABLE S4 The differentially expressed genes in the developmental process of PSC with bile salt strobilization stimulus. The table lists all differentially expressed genes in the PSCs culture with bile salt strobilization stimulus. The statistical comparisons were determined by DESeq.

TABLE S5 The differentially expressed genes in the developmental process of PSC without bile salt strobilization stimulus. The table lists all differentially expressed genes in the PSCs culture without bile salt strobilization stimulus. The statistical comparisons were determined by DESeq.

TABLE S6 The differentially expressed genes between SSD and NSD. The table lists all differentially expressed genes between SSD and NSD. SSD: PSCs culture with bile salt strobilization stimulus; NSD, PSCs culture without bile salt strobilization stimulus.

TABLE S7 Validation of the differentially expressed genes by real-time quantitative PCR. The table lists relative expression levels of 10 differentially expressed gene detected by RNAseq and qPCR. The qPCR fold changes were calculated by the 2^–ΔΔCt^ method.

TABLE S8 Gene Ontology (GO) abundance analysis of the differentially expressed genes in SSD or NSD. The differentially expressed genes and ref represent the number of GO terms in the subgroup or whole gene group, respectively. The hypergeometric distribution test was used to find the significantly different GO term (*P*-value), and the false discovery rate (FDR) was calculated to correct the *P*-value (*Q*-value). SSD: PSCs culture with bile salt strobilization stimulus; NSD: PSCs culture without bile salt strobilization stimulus.

TABLE S9 Comparison of Gene Ontology (GO) terms between SSD and NSD. The table lists the raw reads counts and relative expression levels of all detected mature miRNAs and miRNA stars in the three different life-stages. A statistical comparison was determined by DEGseq (Wang et al., 2010), and the q-value was the correction of the *P*-value with the Benjamini−Hochberg multiple test. PSC, protoscoleces.

TABLE S10 Profiles of differentially expressed miRNAs from the different developmental stages of PSC with or without bile salt strobilization stimulus. The table lists the raw reads counts and relative expression levels of the differentially expressed mature miRNAs and miRNA stars in the different developmental stages of PSC with or without bile salt strobilization stimulus. A statistical comparison was determined by DEGseq (Wang et al., 2010), and the *Q*-value was the correction of the *P*-value with the Benjamini−Hochberg multiple test. The correct *P*-value < 0.001 and fold-change >2.0 were used as the threshold criteria to define significant differences in miRNA expression. PSC, protoscoleces.

TABLE S11 Clustering of the differentially expressed miRNAs from *in vitro* development of PSC. The table lists all differentially expressed miRNAs which have different patterns clustered by the K-means method.

TABLE S12 Validation of the miRNA expression patterns by stem-loop real-time quantitative PCR. The table lists relative expression levels of 10 differentially expressed mature miRNAs and miRNA stars detected by NGS and stem-loop Q-PCR. The fold changes from stem-loop Q-PCR were calculated by the 2^–ΔΔCt^ method.

TABLE S13 Target genes of the differentially expressed miRNAs. The table lists all potential target genes of the differentially expressed miRNAs predicted by Miranda (Enright et al., 2003). The score and energy represent the miRanda score and binding minimum free energy, respectively.

TABLE S14 Nucleotide binding genes which targeted by the differentially expressed miRNAs. The table lists all nucleotide binding genes which regulated by the differentially expressed miRNAs. The score and energy represent the miRanda score and binding minimum free energy, respectively.

TABLE S15 The differentially expressed miRNAs and their targets related with oxidation reduction process The table lists all differentially expressed gene which is related with oxidation reduction, some of them may regulated by the differentially expressed miRNAs.

TABLE S16 Sequences of stem-loop RT primers, forward primers and reverse primer. The table lists all primer sequences used in the qPCR analysis. Normfinder was used to find the most stable expressed house-keeping genes under the test conditions. According to the results obtained, 5.8S rRNA (*M* = 0.08) and GAPDH (*M* = 0.23) were selected as reference genes for qPCR analysis. Furthermore, the PCR amplification efficiencies were calculated using the formula [10^(–1/S)^−1] × 100. Most of these were close to 100% (110%−90%).

## Abbreviations

3′-UTRs: 3′-untranslational regions
AST: aspartate aminotransferase
CE: cystic echinococcosis
DEGs: differentially expressed genes
*E. granulosus*: *Echinococcus granulosus*
FDR: false discovery rate
GO: Gene Ontology
KEGG: Kyoto Encyclopedia of Genes and Genomes
miRNAs: microRNAs
NGS: next generation sequencing technology
NSD: PSC without strobilisation stimulus
PAGE: polyacrylamide gel electrophoresis
PSCs: protoscoleces
SSD: PSC with strobilisation stimulus
TPM: transcripts per million.
