# Supplementary figures and images for: Dynamic Changes in the Global Transcriptome and MicroRNAome Reveal Complex miRNA-mRNA Regulation in Early Stages of the Bi-Directional Development of Echinococcus granulosus Protoscoleces
Source: Front Microbiol. 2020 Apr 9;11:654. doi: 10.3389/fmicb.2020.00654 (PMC7188192; doi:10.3389/fmicb.2020.00654)

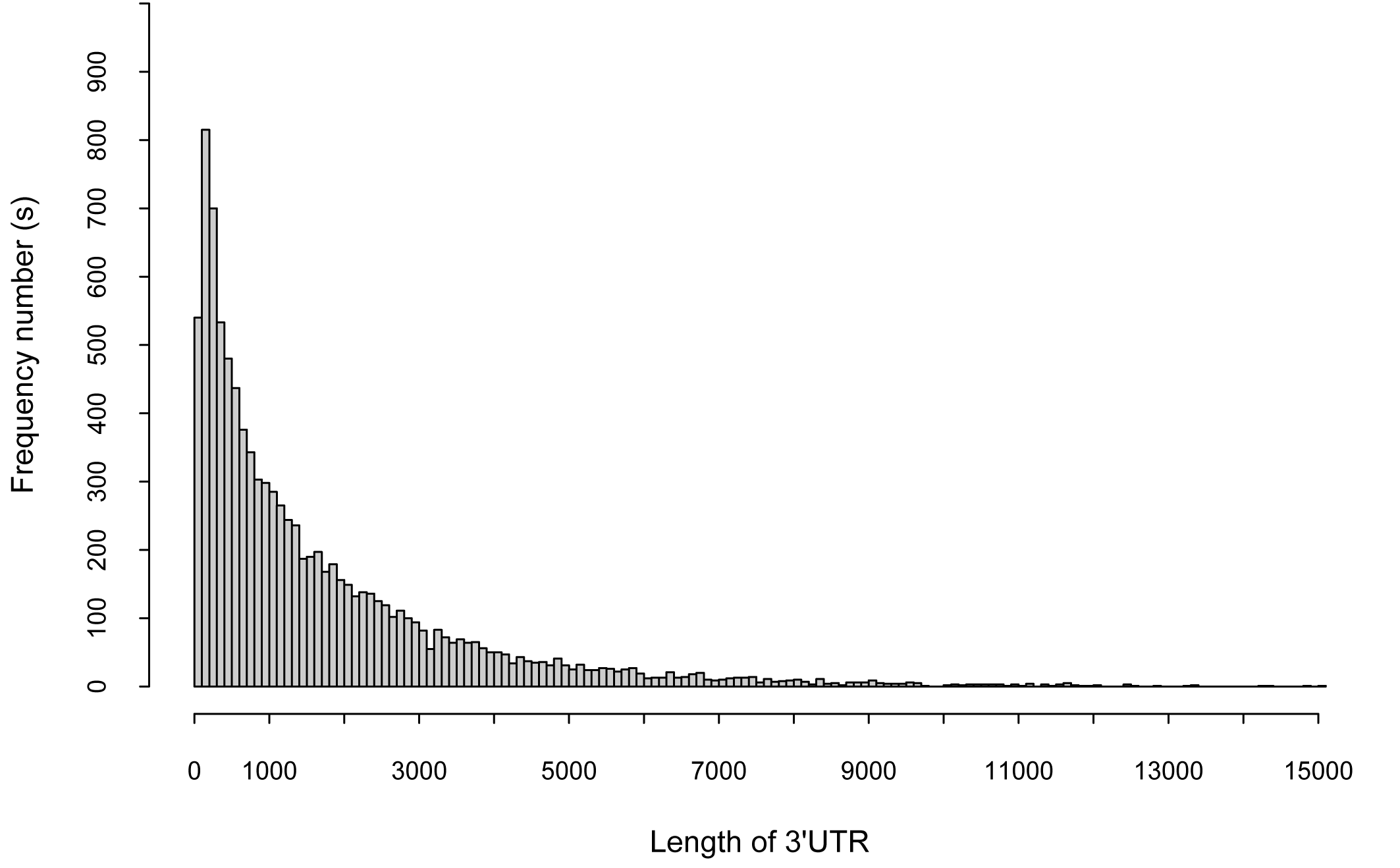

Supplement: FIGURE S1 — Length distribution analysis of E. granulosus 3′-UTRs. The figure summarizes the length distribution of E. granulosus 3′-UTR. A major proportion of the 9,839 3′-UTRs (90.44%) were shorter than 4,000 nucleotides. The median length of the total set of 3′-UTRs was 1,027 nucleotides and the average GC content was 42.45%. [file Image_1.TIF]

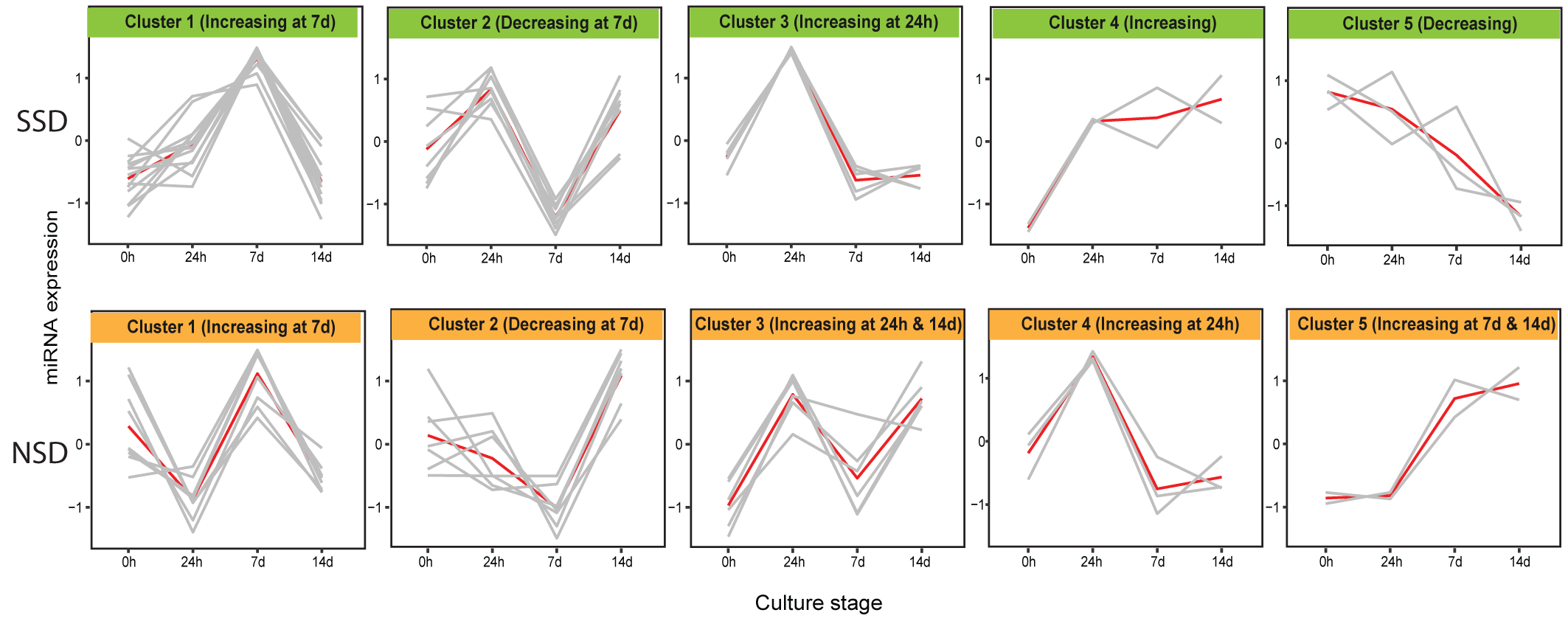

Supplement: FIGURE S2 — Clustering of differentially expressed miRNAs during the in vitro development of PSCs. The differentially expressed miRNAs were clustered using the K-means method. Expression values were normalized and scaled between −1.0 and 1.0 (Y-axis). The X-axis indicates the time-points when RNA was subjected to transcriptome sequencing. A description of the pattern of expression belonging to each cluster is shown for each panel. [file Image_2.TIF]

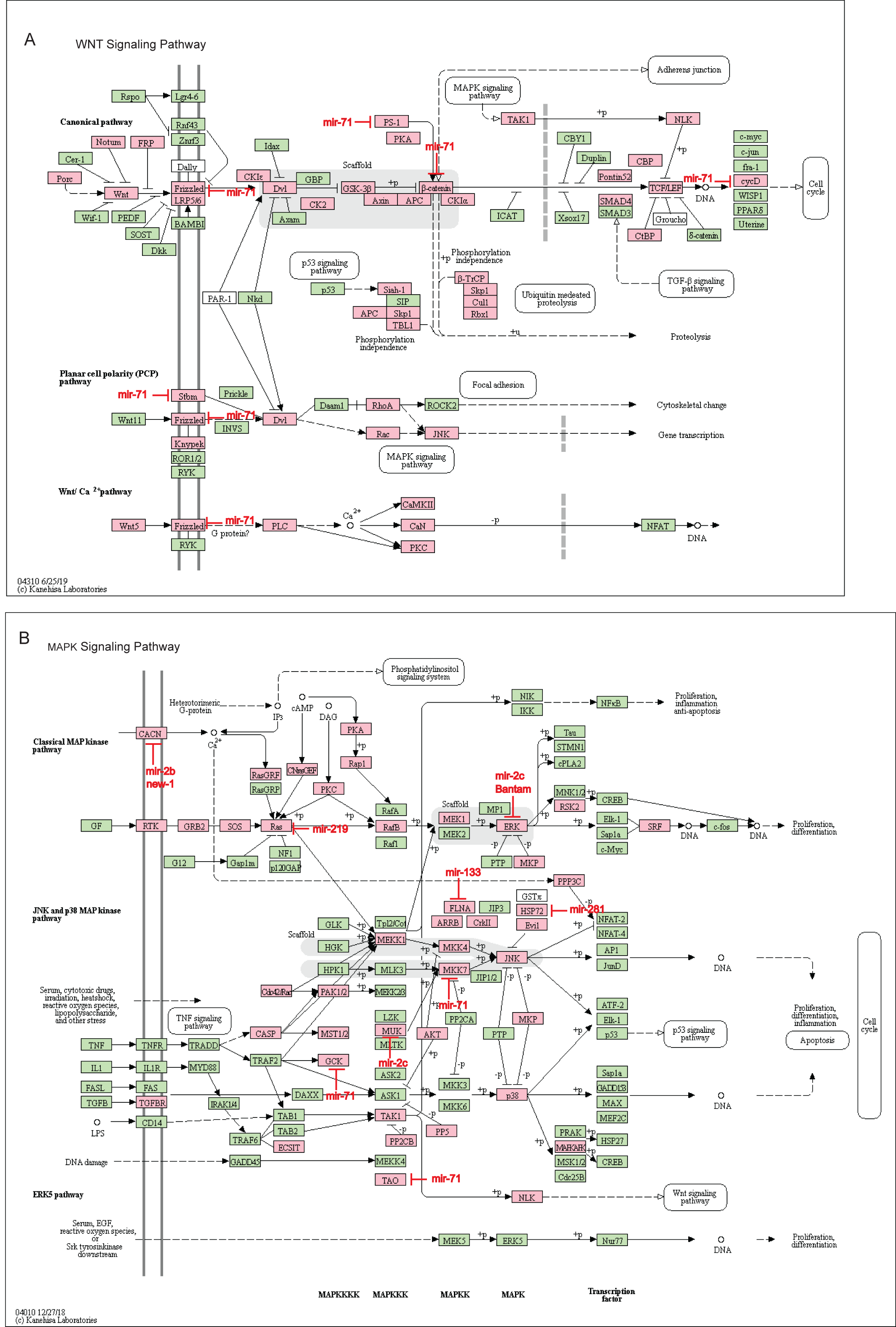

Supplement: FIGURE S3 — Predicted targets of the differentially expressed miRNAs within the context of selected signaling pathways. (A) Wnt signaling pathway; (B) MAPK signaling pathway. Pink boxes represent gene orthologs present in E. granulosus. miRNAs that target genes in these pathways are shown in red. [file Image_3.TIF]
